# Efficacy of Low-Carbohydrate Diets Versus Low-Fat Diets in Glycemic Control Among Patients With Type 2 Diabetes: A Systematic Review

**DOI:** 10.7759/cureus.77004

**Published:** 2025-01-06

**Authors:** Delphyne Anyang Kaakyire, Omar O Abdelfattah, Aroon Kumar, Sami Qadeer

**Affiliations:** 1 Internal Medicine, Saint Petersburg State University, Saint Petersburg, RUS; 2 Internal Medicine, Heliopolis Hospital, Cairo, EGY; 3 Medicine and Surgery, Khairpur Medical College, Khairpur, PAK; 4 Internal Medicine, Nishtar Medical University, Multan, PAK

**Keywords:** diabetes medication, dietary interventions, glycemic control, hba1c, lipid profiles, low-carbohydrate diet, low-fat diet, randomized controlled trials, type 2 diabetes, weight management

## Abstract

This systematic review evaluates the comparative efficacy of low-carbohydrate diets (LCDs) versus low-fat diets (LFDs) in improving glycemic control, weight management, and lipid profiles in individuals with type 2 diabetes mellitus (T2DM) or prediabetes. Seven randomized controlled trials involving diverse populations were included, with dietary interventions ranging from very low-carbohydrate ketogenic (LCK) diets (typically <10% of total caloric intake from carbohydrates, with higher fat and moderate protein) to moderate carbohydrate regimens (30-45% of total calories). LFDs, in contrast, prioritized carbohydrate intake (50-60% of total calories), with reduced fat (<20-30%) and moderate protein (15-20%). Across studies, LCDs consistently demonstrated greater reductions in HbA1c, fasting glucose, and triglycerides, alongside superior weight loss and increased high-density lipoprotein cholesterol compared to LFDs. Additionally, LCDs were associated with significant reductions in diabetes medication use, highlighting their potential to decrease pharmacological dependency and improve metabolic outcomes, including enhanced insulin sensitivity and reduced inflammation. Despite variability in long-term outcomes and adherence, LCDs emerged as a promising alternative to traditional dietary approaches for managing T2DM. Further research is warranted to explore strategies to improve dietary adherence, such as behavioral interventions and technological support, and to evaluate long-term sustainability, including their effects on cardiovascular health and quality of life. These findings underscore the transformative potential of LCDs in diabetes management and highlight the need for personalized dietary approaches.

## Introduction and background

Type 2 diabetes mellitus (T2DM) is a chronic metabolic disorder characterized by hyperglycemia resulting from impaired insulin secretion, insulin resistance, or a combination of both [1,2]. Its prevalence has been steadily increasing, with significant implications for global health due to its association with severe complications, including cardiovascular diseases, nephropathy, neuropathy, and retinopathy [3,4]. Effective glycemic control is critical in reducing the burden of these complications, and dietary interventions have emerged as cornerstone strategies in diabetes management. Among these, low-carbohydrate diets (LCDs) and low-fat diets (LFDs) are widely studied for their potential to improve glycemic control, body weight, and overall metabolic health [5]. LCDs are typically structured to limit carbohydrate intake to less than 10-30% of total daily calories, often emphasizing higher proportions of fat (40-60%) and moderate protein (20-30%). In contrast, LFDs aim to restrict fat intake to less than 20-30% of total daily calories, prioritizing carbohydrates (50-60%) as the primary energy source and maintaining moderate protein intake (15-20%). These distinct macronutrient compositions influence glycemic control and metabolic outcomes differently, contributing to the ongoing debate regarding their comparative efficacy in managing glycemic levels and reducing diabetes-related risks. This systematic review aims to synthesize current evidence on the efficacy of LCDs versus LFDs in glycemic control among patients with T2DM, providing insights into their relative effectiveness to guide clinical and dietary recommendations.

The PICO framework forms the foundation of this systematic review, structuring the research question and guiding the selection and evaluation of studies [6]. The Population (P) comprises adult patients diagnosed with T2DM, ensuring that the findings are relevant to the population most affected by the disease. The Intervention (I) focuses on LCDs, which emphasize reducing carbohydrate intake to improve glycemic control by minimizing postprandial glucose spikes and enhancing insulin sensitivity. The Comparison (C) involves LFDs, traditionally recommended for cardiovascular health and weight management, to evaluate their relative efficacy in glycemic control. The Outcome (O) includes key measures of glycemic control, such as hemoglobin A1c (HbA1c), fasting plasma glucose, postprandial glucose levels, and insulin sensitivity, alongside secondary outcomes such as weight loss and lipid profile changes. This PICO framework ensures a systematic approach to identifying and synthesizing high-quality evidence, enabling a focused and clinically meaningful comparison of these dietary interventions.

## Review

Materials and methods

Search Strategy

The search strategy for this systematic review was designed to comprehensively identify studies evaluating the efficacy of LCDs versus LFDs in managing glycemic control among individuals with T2DM. A structured approach was employed to search major databases, including PubMed, MEDLINE, and Cochrane Library, using keywords such as "low-carbohydrate diet", "low-fat diet", "type 2 diabetes", "HbA1c", and "glycemic control". Boolean operators and database-specific filters were applied to refine results and ensure relevance. The inclusion criteria focused on randomized controlled trials (RCTs) published in peer-reviewed journals, conducted in adults with T2DM or prediabetes, and reporting outcomes such as HbA1c, fasting glucose, weight loss, or lipid profiles. The Preferred Reporting Items for Systematic Reviews and Meta-Analyses (PRISMA) guidelines were adhered to throughout the review process, ensuring a transparent and methodologically rigorous approach to study selection, data extraction, and synthesis [7]. This robust strategy maximized the comprehensiveness and reliability of the evidence included in the review.

Eligibility Criteria

The eligibility criteria for this systematic review were carefully designed to ensure the inclusion of high-quality studies that directly addressed the research question [8]. Only RCTs were considered, given their methodological rigor in minimizing biases and establishing causal relationships. Studies were required to focus on adults diagnosed with T2DM or prediabetes, ensuring the relevance of the findings to this specific population. Interventions included LCDs with varying carbohydrate intake thresholds, while the comparison group followed LFDs or standard dietary recommendations. Outcomes of interest encompassed key measures of glycemic control, such as HbA1c, fasting plasma glucose, and glycemic variability, as well as secondary outcomes such as weight loss, lipid profile changes, and reductions in diabetes medication use. Studies had to report these outcomes quantitatively to facilitate accurate data extraction and analysis.

To maintain the relevance and reliability of the evidence, studies were excluded if they involved participants with comorbid conditions that could confound the dietary effects on glycemic control, such as advanced cardiovascular disease, chronic kidney disease, or uncontrolled hypertension, as these conditions could independently affect glycemic outcomes and response to dietary interventions. Trials were also excluded if they focused solely on short-term interventions of less than three months or lacked a clear definition of dietary protocols, as these factors could compromise the reliability and interpretability of the findings. Regarding inclusion criteria, studies that recruited adult participants (aged 18 years and above) with a confirmed diagnosis of T2DM or prediabetes were included, without restrictions on gender, ethnicity, or baseline BMI, to ensure broad applicability of the findings. However, studies exclusively targeting pediatric populations or pregnant individuals were excluded, as these groups have unique physiological and dietary considerations. Non-English language studies, grey literature, and conference abstracts were also omitted to prioritize peer-reviewed, full-text articles. This carefully designed eligibility framework ensured a robust and focused selection of studies, facilitating a comprehensive and meaningful synthesis of the evidence on the comparative efficacy of LCDs and LFDs in managing T2DM.

Data Extraction

Data extraction for this systematic review was conducted using a standardized process to ensure consistency and accuracy across all included studies. Key details such as study design, sample size, population characteristics, intervention and comparison protocols, duration, and outcomes were systematically extracted. Specific data points included baseline and follow-up measures of HbA1c, fasting glucose, weight, lipid profiles, and medication use, along with statistical details such as mean differences, confidence intervals, and p-values. Clinically significant changes were defined as reductions in HbA1c of at least 0.5%, which has been associated with a meaningful decrease in the risk of diabetes-related complications, and fasting glucose changes of 10 mg/dL or more, indicative of improved glycemic control. The extraction process also accounted for intervention adherence, dropout rates, and any reported adverse effects. Multiple reviewers independently extracted data to minimize errors and biases, with discrepancies resolved through discussion or consultation with a third reviewer. This robust approach allowed for a comprehensive and reliable synthesis of the evidence, ensuring that findings align with practical clinical implications.

Data Analysis and Synthesis

The data analysis and synthesis for this systematic review focused on evaluating the comparative efficacy of LCDs and LFDs in managing glycemic control among individuals with T2DM. Key outcomes, such as changes in HbA1c, fasting glucose, weight, and lipid profiles, were synthesized using descriptive statistics and reported effect sizes, including confidence intervals and p-values, where available. Given the variability in dietary protocols and study designs, a narrative synthesis approach was employed to integrate findings and identify patterns across studies. Results were stratified by intervention type and outcome category to provide a clear comparison of LCD and LFD effects. Studies reporting reductions in medication use and long-term adherence to diets were highlighted to emphasize practical implications. This systematic approach ensured a comprehensive interpretation of the data, while adhering to PRISMA guidelines for methodological transparency and rigor.

Results

Study Selection Process

**Figure 1:**
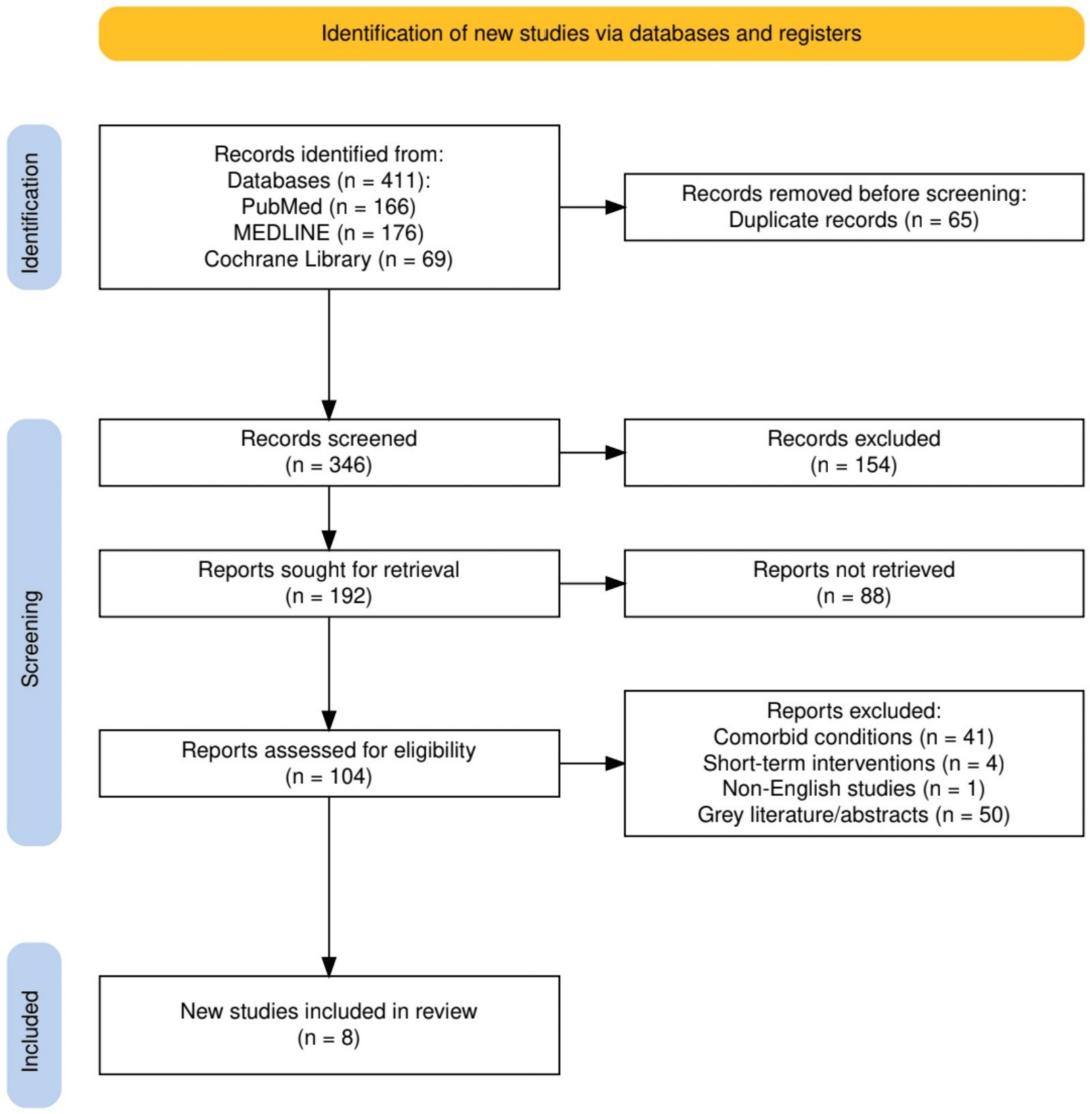
PRISMA flowchart representing the study selection process. PRISMA, Preferred Reporting Items for Systematic Reviews and Meta-Analyses

The study selection process adhered to a systematic and rigorous approach, as outlined in Figure 1. Initially, 411 records were identified through comprehensive searches across databases such as PubMed (166 records), MEDLINE (176 records), and the Cochrane Library (69 records). After removing 65 duplicates, 346 records were screened based on title and abstract. Of these, 154 records were excluded for failing to meet the inclusion criteria. Full-text retrieval was sought for 192 reports, of which 88 could not be retrieved. A total of 104 reports underwent detailed eligibility assessment, resulting in the exclusion of studies due to reasons such as comorbid conditions (41 reports), short-term interventions (four reports), non-English language (one report), and grey literature or conference abstracts (50 reports). Ultimately, eight studies met the eligibility criteria and were included in the final review. This stepwise process ensured the inclusion of high-quality studies relevant to the research question.

Characteristics of the Selected Studies

Table 1 provides an overview of the key characteristics, interventions, and outcomes of the studies included in this review. It summarizes the population, dietary interventions, comparisons, and primary findings, highlighting the evidence supporting the efficacy of LCDs compared to LFDs in managing T2DM. This comprehensive synthesis facilitates a clearer understanding of the comparative effects of these dietary approaches on glycemic control, weight loss, and metabolic health.

**Table 1:** Summary of all the selected articles in the systematic review. ADA, American Diabetes Association; BMI, body mass index; CI, confidence interval; E, units of insulin (symbol often used in insulin dosing); HbA1c, hemoglobin A1c (glycated hemoglobin); HC, high-carbohydrate (diet); HDL, high-density lipoprotein (cholesterol); LC, low-carbohydrate (diet); LCD, low-carbohydrate diet; LCK, low-carbohydrate ketogenic (diet); LFD, low-fat diet; MCCR, moderate-carbohydrate, calorie-restricted (diet); mg/dL, milligrams per deciliter (unit of concentration); mmol/L, millimoles per liter (unit of concentration); p-value, probability value (statistical significance measure); T2DM, type 2 diabetes mellitus

Study	Population	Intervention	Comparison	Outcomes	Statistical Data	Conclusion
Dorans et al., 2022 [9]	150 participants aged 40-70 years with untreated HbA1c (6.0%-6.9%), 72% women, 59% Black.	Low-carbohydrate diet (<40 g carbs/day for 3 months; <60 g for months 3-6) with dietary counseling.	Usual diet (no specific dietary counseling).	Greater 6-month reductions in HbA1c (-0.23%), fasting glucose (-10.3 mg/dL), and body weight (-5.9 kg).	HbA1c: net difference -0.23% (95% CI, -0.32% to -0.14%, p < 0.001); fasting glucose: -10.3 mg/dL (95% CI, -15.6 to -4.9 mg/dL, p < 0.001); weight: -5.9 kg (95% CI, -7.4 to -4.4 kg, p < 0.001).	LCD improved glycemia and weight; useful for preventing and treating T2DM, but effects independent of weight loss need further evaluation.
Saslow et al., 2017 [10]	34 overweight adults (BMI > 25) with HbA1c > 6.0%, randomized to very LCD (n=16) or moderate-carb (n=18) diet	Very LCK diet; encouraged physical activity, adequate sleep, and mindful eating.	MCCR diet with similar lifestyle recommendations.	Greater 12-month reductions in HbA1c (-0.5% with LCK vs. -0.2% with MCCR, p=0.007), weight loss (-7.9 kg with LCK vs. -1.7 kg with MCCR, p<0.001), and medication use.	HbA1c: baseline 6.6% to 6.1% (LCK) vs. 6.9% to 6.7% (MCCR), p=0.007. Weight: baseline 99.9 kg to 92.0 kg (LCK) vs. 97.5 kg to 95.8 kg (MCCR), p<0.001.	LCK diet led to significantly greater reductions in HbA1c, weight, and medication use compared to MCCR over 12 months.
Tay et al., 2015 [11]	115 obese adults with T2D (mean age 58 ± 7 years, BMI 34.6 ± 4.3, HbA1c 7.3 ± 1.1%, duration of diabetes 8 ± 6 years).	Very LCD: 14% carbs (<50 g/day), 28% protein, 58% fat (<10% saturated fat); supervised aerobic and resistance exercise (60 min, 3 days/week).	HC diet: 53% carbs, 17% protein, 30% fat (<10% saturated fat); same exercise protocol.	Both groups: similar weight loss (-9.8 kg LC vs. -10.1 kg HC) and HbA1c reduction (-1.0% in both groups). LCD achieved better glycemic variability, HDL, triglycerides, and medication reduction.	HbA1c: -1.0% in both groups (p ≥ 0.10). Glycemic variability: -0.5 mmol/L LC vs. -0.05 mmol/L HC (p = 0.003). HDL: +0.1 mmol/L LC vs. +0.06 mmol/L HC (p = 0.002). Triglycerides: -0.4 mmol/L LC vs. -0.01 mmol/L HC (p = 0.001).	LCD improved lipid profile, glycemic stability, and reduced medication needs more effectively than HC diet, making it a promising approach for T2D management.
Guldbrand et al., 2012 [12]	61 adults with T2D (mean BMI 32.7 ± 5.4 kg/m², HbA1c 57.0 ± 9.2 mmol/mol) recruited from primary care.	LCD: 20% energy from carbohydrates; compliance supported by four group meetings.	LFD: 55-60% energy from carbohydrates; compliance supported by four group meetings.	Similar weight loss at 6 months (-4.31 kg LCD vs. -3.99 kg LFD, p < 0.001 within groups). LCD improved HbA1c (-4.8 mmol/mol at 6 months, p = 0.004). HDL increased with LCD (1.13 to 1.25 mmol/L, p = 0.018). Insulin doses reduced significantly in LCD group.	HbA1c: LCD -4.8 ± 8.3 mmol/mol at 6 months (p = 0.004); Weight loss: LCD -4.31 ± 3.6 kg vs. LFD -3.99 ± 4.1 kg at 6 months (p < 0.001 within groups); HDL: LCD +0.12 mmol/L (p = 0.018); Insulin: LCD reduced to 30 ± 47 E vs. LFD 38 ± 48 E at 6 months (p = 0.046).	LCD resulted in similar weight loss but greater reductions in HbA1c, insulin use, and improved HDL compared to LFD. Aiming for 20% energy from carbs is a safe and effective alternative.
Davis et al., 2009 [13]	105 overweight adults with T2D in a 1-year randomized clinical trial. Outcomes measured at 3, 6, and 12 months.	LCD: Emphasized reduced carbohydrate intake for weight loss and glycemic control.	LFD: focused on reduced fat intake for weight loss and glycemic control.	Faster weight loss in LC group at 3 months (p = 0.005), but similar weight reduction (3.4%) in both groups at 1 year. No significant change in HbA1C at 1 year. Greater HDL increase in LC group (p = 0.002).	Weight: faster loss in LC group at 3 months (p = 0.005). HDL: greater increase in LC group (p = 0.002). No significant HbA1C change in either group at 1 year.	LCD achieved faster weight loss initially and greater HDL increases, but 1-year weight and HbA1C outcomes were similar to the LFD.
Saslow et al., 2017 [14]	25 overweight adults with T2D (BMI ≥ 25, HbA1c 6.5%-9.0%) randomized into an online intervention or control group.	Ad libitum very LCK diet with lifestyle recommendations (physical activity, sleep, mindfulness) delivered online.	Online diet program based on the ADA's “Create Your Plate” method.	Greater HbA1c reduction in the intervention group (-0.8% vs. -0.3%, p = 0.002), more weight loss (-12.7 kg vs. -3.0 kg, p < 0.001), and greater triglyceride reduction (-60.1 mg/dL vs. -6.2 mg/dL, p = 0.01). 90% lost ≥5% body weight in the intervention group vs. 29% in the control group (p = 0.01).	HbA1c: -0.8% (95% CI -1.1% to -0.6%) intervention vs. -0.3% (95% CI -0.6% to 0.0%) control, p = 0.002. Weight loss: -12.7 kg (95% CI -16.1 to -9.2 kg) intervention vs. -3.0 kg (95% CI -7.3 to 1.3 kg) control, p < 0.001.	The online very LCK diet program led to better glycemic control, weight loss, and triglyceride reduction compared to the control, highlighting the potential for wider adoption.
Iqbal et al., 2010 [15]	144 obese diabetic participants randomized to LCD or LFD, with a 24-month low-intensity intervention.	LCD (<30 g/day) with weekly group sessions for the first month, then monthly sessions for 24 months.	LFD (≤30% calories from fat, 500 kcal/day deficit) with the same session frequency.	At 24 months, the LCD group lost 1.5 kg vs. 0.2 kg in the LFD group (p = 0.147). No significant differences in lipids, glycemic indices, or dietary intake between groups at any time point.	Weight loss at 24 months: LCD -1.5 kg vs. LFD -0.2 kg (p = 0.147). No significant differences in glycemic indices or lipids (p > 0.05).	Low-intensity LCD intervention resulted in modest weight loss but showed no significant advantages over LFD for glycemic or lipid outcomes at 24 months.
Saslow et al., 2014 [16]	34 overweight or obese adults with T2D or prediabetes (HbA1c > 6%), 74% on oral diabetes medications.	Very LCK diet: high fat, non-calorie-restricted, aimed to induce nutritional ketosis, with 13 sessions over 3 months.	Medium carbohydrate, low fat, calorie-restricted, carbohydrate-counting diet (MCCR), consistent with ADA guidelines.	LCK group: HbA1c reduced by 0.6% (95% CI, -1.1% to -0.03%, p = 0.04), 44% discontinued diabetes medications (p = 0.03), lost 5.5 kg vs. 2.6 kg in the MCCR group (p = 0.09).	HbA1c: LCK -0.6% (p = 0.04) vs. no change in MCCR. Medication discontinuation: 44% in LCK vs. 11% in MCCR (p = 0.03). Weight loss: LCK -5.5 kg vs. MCCR -2.6 kg (p = 0.09).	LCK diet improved glycemic control and facilitated significant reductions in diabetes medication use compared to MCCR, suggesting a promising dietary approach.

Each study was evaluated using the Cochrane Risk of Bias Tool, focusing on key methodological aspects such as randomization, blinding, adherence, outcome reporting, and potential biases. The quality assessment categorizes studies as high, moderate, or low based on their methodological rigor and the reliability of their findings. This evaluation provides a clear understanding of the strengths and limitations of the included evidence, ensuring transparency and aiding in the interpretation of the review's overall findings. Table 2 provides the risk-of-bias assessment of each study.

**Table 2:** The risk-of-bias assessment for included studies.

Study	Randomization	Blinding	Adherence	Outcome Reporting	Attrition Bias	Overall Risk of Bias
Dorans et al., 2022 [9]	Randomized; well-described method	Not blinded; potential performance bias	Moderate; diet adherence not objectively monitored	Robust; statistical reporting clear	Low dropout rates reported	Moderate
Saslow et al., 2017 [10]	Randomized; clear allocation	Not blinded; adherence self-reported	Moderate; potential bias in adherence reporting	Complete; all planned outcomes reported	Small sample size, potential bias in representativeness	Moderate
Tay et al., 2015 [11]	Randomized; allocation method detailed	Not blinded; potential bias in adherence reporting	High adherence; supervised exercise program	Detailed and transparent	Low attrition bias due to regular follow-up	Low
Guldbrand et al., 2012 [12]	Randomized; clear description	Not blinded; potential bias in outcome assessment	Moderate; group meetings supported compliance	Comprehensive; key outcomes clearly reported	Small sample size, some missing adherence data	Moderate
Davis et al., 2009 [13]	Randomized; robust method	Not blinded; adherence self-reported	Moderate; adherence variability reported	Complete; robust reporting of outcomes	Moderate dropout rate, potential bias	Moderate
Saslow et al., 2017 [14]	Randomized; allocation described	Not blinded; reliance on online adherence reporting	Low; remote intervention challenges adherence	Partial; focus on primary outcomes	Small sample size, high potential for attrition	High
Iqbal et al., 2010 [15]	Randomized; allocation unclear	Not blinded; adherence variability noted	Low; limited adherence support provided	Incomplete; secondary outcomes not reported	High attrition over 24 months	High
Saslow et al., 2014 [16]	Randomized; allocation described	Not blinded; adherence self-reported	Moderate; lifestyle components variably adhered	Detailed; all planned outcomes reported	Small sample size, short duration	Moderate

Discussion

This systematic review demonstrates that LCDs consistently outperform LFDs in improving glycemic control, weight management, and lipid profiles among patients with T2DM. Across the included studies, LCDs showed significant reductions in HbA1c, fasting glucose, and triglycerides, alongside superior weight loss and increased high-density lipoprotein (HDL) cholesterol. These findings highlight the potential of LCDs as an effective dietary approach to enhance metabolic outcomes and reduce medication dependence in T2DM management.

The findings from the studies consistently demonstrate the potential of LCDs to improve glycemic control, weight management, and other metabolic parameters in individuals with T2DM or prediabetes. Across the studies, LCDs were shown to significantly reduce HbA1c levels, a critical marker of glycemic control. For instance, Dorans et al. [9] reported a significant HbA1c reduction of -0.23% (95% CI, -0.32% to -0.14%; p < 0.001) over six months, with concurrent improvements in fasting glucose (-10.3 mg/dL; p < 0.001) and weight loss (-5.9 kg; p < 0.001). Similarly, Saslow et al. [10] observed a greater reduction in HbA1c for participants following a very LCK diet compared to a moderate-carbohydrate diet (LCK: -0.5% vs. MCCR: -0.2%; p = 0.007). LCK diets are typically structured with carbohydrate intake limited to less than 10% of total daily calories (approximately 20-50 g/day), with fat comprising 70-80% and protein 15-20% of total caloric intake, designed to induce a state of nutritional ketosis. In contrast, moderate-carbohydrate diets generally allow for 30-45% of total daily caloric intake from carbohydrates, with reduced fat intake and moderate protein levels, often adhering to calorie restrictions for weight management. The same study highlighted significant weight loss in the LCK group (-7.9 kg vs. -1.7 kg; p < 0.001) and a substantial reduction in diabetes medication use, further underscoring the diet's multifaceted benefits.

Additional studies supported these findings while highlighting unique aspects of LCD efficacy. Tay et al. [11] found comparable reductions in HbA1c for both LCD and high-carbohydrate diet (-1.0%, p ≥ 0.10) but noted greater improvements in glycemic variability (-0.5 mmol/L vs. -0.05 mmol/L; p = 0.003) and triglyceride levels (-0.4 mmol/L vs. -0.01 mmol/L; p = 0.001) in the LCD group. Guldbrand et al. also demonstrated a significant reduction in HbA1c in the LCD group (-4.8 mmol/mol; p = 0.004) alongside an improvement in HDL cholesterol (LCD: +0.12 mmol/L; p = 0.018) and reduced insulin doses (p = 0.046). These findings align with those reported by Davis et al. [13], where initial weight loss was faster in the low-carb group (p = 0.005), and HDL cholesterol improvements were significantly greater (p = 0.002). Collectively, the studies underscore the effectiveness of LCDs in addressing glycemic control and metabolic health, making them a compelling option for managing T2DM.

The findings from this systematic review align closely with previous research, which underscores the efficacy of LCDs in improving glycemic control and metabolic health in individuals with T2DM [17]. Consistent with earlier studies, LCDs demonstrated significant reductions in HbA1c and weight, as well as improvements in lipid profiles, such as increased HDL cholesterol and reduced triglycerides. For example, Tay et al.’s findings [11] corroborate prior findings that LCDs can improve glycemic variability and triglyceride levels more effectively than high-carbohydrate diets. Similarly, the reductions in diabetes medication use observed by Saslow et al. [10] and Guldbrand et al. [12] mirror earlier evidence suggesting that LCDs can reduce reliance on pharmacological interventions. However, discrepancies emerge in the long-term efficacy of LCDs, as seen in Davis et al.’s study [13], which reported no significant differences in HbA1c between LCD and LFD after one year. These differences may stem from variations in study design, such as the intensity and duration of dietary interventions or the level of support provided to participants. Dietary adherence likely played a key role, as sustaining strict carbohydrate restrictions over an extended period can be challenging. Furthermore, differences in participants' metabolic responses, baseline glycemic levels, or the presence of comorbid conditions may have influenced outcomes. These findings reinforce the growing consensus on the utility of LCDs while emphasizing the need for personalized dietary strategies and further research to address variations in adherence and long-term outcomes [18,19].

The strengths of this systematic review lie in its comprehensive inclusion of RCTs that employed robust methodologies, such as clear intervention protocols, appropriate comparison groups, and the use of clinically relevant outcomes such as HbA1c, weight loss, and lipid profiles. The consistent adherence to standardized measures across studies enhances the reliability and comparability of findings. Additionally, the use of long-term interventions in several studies, such as those by Tay et al. [11] and Iqbal et al. [15], provides valuable insights into the sustainability of LCDs. However, the review is not without limitations. Variability in sample sizes, with some studies having relatively small cohorts, such as the study by Saslow et al. [16], may limit the generalizability of the results. Differences in intervention intensities and participant adherence also introduce potential biases. Furthermore, most studies relied on self-reported dietary intake, which may not accurately reflect compliance. Despite these constraints, the consistency of significant findings across diverse populations and methodologies supports the validity of the conclusions and emphasizes the need for further large-scale studies to address these limitations.

The findings of this systematic review carry significant implications for clinical practice, public health policy, and future research in managing T2DM. The demonstrated efficacy of LCDs in improving glycemic control, reducing body weight, and enhancing lipid profiles suggests that these dietary interventions should be considered as a viable alternative or adjunct to traditional LFDs in diabetes management guidelines [20,21]. Clinicians may leverage these findings to tailor dietary recommendations to individual patients, emphasizing the potential for reduced reliance on pharmacological interventions and improved metabolic outcomes. From a policy perspective, incorporating LCD-focused nutritional education into public health programs could empower patients to adopt sustainable dietary changes [22]. LFDs, in comparison, are typically structured to limit fat intake to less than 20-30% of total daily calories, prioritizing carbohydrates (50-60%) as the primary energy source, with moderate protein intake (15-20%). These diets are often designed to reduce caloric density and are traditionally recommended for cardiovascular health and weight management. The review also highlights the need for future research to explore long-term adherence strategies, refine dietary protocols for different demographic groups, and investigate the role of LCDs in preventing diabetes-related complications [23]. By bridging these gaps, the findings of this review can serve as a foundation for advancing personalized nutrition and evidence-based diabetes management strategies.

Future research should focus on addressing several key gaps identified in this review to enhance our understanding of the long-term efficacy and applicability of LCDs in managing T2DM [24]. One critical area is the need for large-scale, multicenter RCTs with diverse populations to improve the generalizability of findings. Studies should also investigate the sustainability of LCDs over extended periods, exploring strategies to improve adherence and assessing their impact on long-term glycemic control and complication rates. Additionally, research examining the physiological mechanisms underlying LCDs' effects, such as their role in modulating insulin sensitivity and inflammation, could provide deeper insights into their benefits [25]. Comparative studies that evaluate LCDs against other dietary interventions, including plant-based or Mediterranean diets, would help identify optimal dietary patterns for various subgroups. Lastly, integrating advanced technologies such as continuous glucose monitoring and machine learning to tailor LCD recommendations could pave the way for precision nutrition in diabetes management, addressing individual variability in response to dietary changes.

## Conclusions

This systematic review underscores the potential of LCDs as an effective dietary intervention for managing T2DM. The findings consistently demonstrate significant improvements in glycemic control, weight management, and lipid profiles, with reductions in medication dependence observed in several studies. These results highlight the utility of LCDs not only as a viable alternative to traditional LFDs but also as a personalized approach to addressing the diverse metabolic needs of individuals with T2DM. While variability in long-term outcomes and adherence emphasizes the need for further research, this review contributes valuable evidence supporting the integration of LCDs into clinical practice and public health strategies. By providing a foundation for future exploration, it advances the conversation on precision nutrition and the optimization of dietary interventions for diabetes management.
